# Glutathione Enhances Auxin Sensitivity in Arabidopsis Roots

**DOI:** 10.3390/biom10111550

**Published:** 2020-11-13

**Authors:** Taras Pasternak, Klaus Palme, Ivan A. Paponov

**Affiliations:** 1 Institute of Biology II/Molecular Plant Physiology, Centre for BioSystems Analysis, BIOSS Centre for Biological Signalling Studies University of Freiburg, 79104 Freiburg, Germany; klaus.palme@biologie.uni-freiburg.de; 2 Department of Food Science, Aarhus University, 8200 Aarhus N, Denmark

**Keywords:** auxin, auxin response, glutathne (GSH), auxin transport, root apical meristem, lateral root formation, auxin biosynthesis

## Abstract

Root development is regulated by the tripeptide glutathione (GSH), a strong non-enzymatic antioxidant found in plants but with a poorly understood function in roots. Here, Arabidopsis mutants deficient in GSH biosynthesis (*cad2*, *rax1*, and *rml1*) and plants treated with the GSH biosynthesis inhibitor buthionine sulfoximine (BSO) showed root growth inhibition, significant alterations in the root apical meristem (RAM) structure (length and cell division), and defects in lateral root formation. Investigation of the molecular mechanisms of GSH action showed that GSH deficiency modulated total ubiquitination of proteins and inhibited the auxin-related, ubiquitination-dependent degradation of Aux/IAA proteins and the transcriptional activation of early auxin-responsive genes. However, the DR5 auxin transcriptional response differed in root apical meristem (RAM) and pericycle cells. The RAM DR5 signal was increased due to the up-regulation of the auxin biosynthesis TAA1 protein and down-regulation of PIN4 and PIN2, which can act as auxin sinks in the root tip. The transcription auxin response (the DR5 signal and expression of auxin responsive genes) in isolated roots, induced by a low (0.1 µM) auxin concentration, was blocked following GSH depletion of the roots by BSO treatment. A higher auxin concentration (0.5 µM) offset this GSH deficiency effect on DR5 expression, indicating that GSH deficiency does not completely block the transcriptional auxin response, but decreases its sensitivity. The ROS regulation of GSH, the active GSH role in cell proliferation, and GSH cross-talk with auxin assume a potential role for GSH in the modulation of root architecture under stress conditions.

## 1. Introduction

The continuous adjustment of phenotypic responses is a vital component of the process of plant adaptation to specific growth conditions and involves the coordinated activation of multiple cellular sensors and signalling pathways (Potters et al., 2007, 2009) [1,2]. One of the prominent candidates for these sensing events is glutathione (γ-l-glutamyl-l-cysteinyl-glycine, GSH). GSH is a ubiquitous tripeptide that takes part in transducing environmental signals and plays a key role in plant responses to abiotic stress [3].

GSH possesses a cysteine thiol group that serves as a proton donor; therefore, GSH can undergo reversible oxidation to form glutathione di-sulfide (GSSG), which, in turn, can be reduced back to GSH through the activity of glutathione reductase (GR). This alternation of glutathione redox states contributes to the maintenance of the redox balance of ascorbate (ASC) or glutaredoxin/thioredoxin (reviewed in [4,5,6,7]). Typically, cells in non-stress conditions have a high GSH/GSSG (2GSH) ratio, which is maintained through de novo GSH synthesis, enzymatic reduction of GSSG by GR, and uptake of exogenous GSH [8].

In Arabidopsis, the contribution of these three pathways in the maintenance of GSH pools depends on the developmental stage and occurs in a cell-specific manner. In particular, GSSG reduction is essential during the early stages of seed germination, when the GSH pool is mainly represented by plastid GSSG. By contrast, de novo biosynthesis becomes crucial at 4 days after germination. GSH biosynthesis has been extensively studied and is initiated by two sequential ATP-dependent reactions occurring between glutamate, cysteine, and glycine. The reactions are catalyzed by γ-glutamyl cysteine synthetase (γ-ECS), the rate-limiting step, followed by glutathione synthetase [9,10,11], respectively.

The role of cellular glutathione homeostasis in the detoxification of reactive oxygen species [12,13] and of xenobiotic compounds [14] is well documented, as its function in general stress tolerance [15]. Other roles of GSH include the maintenance of a pool of reduced ascorbate as a part of the ascorbate-glutathione cycle [12], DNA synthesis, the modulation of various enzyme activities [16,17,18] and sulfur storage and transport [19,20]. GSH has also been implicated in the regulation of the cell cycle [21,22,23,24], cell elongation [25], and, in white spruce, of somatic embryogenesis [26,27,28].

Genetic studies with GSH-deficient mutants and chemical application of the GSH biosynthesis inhibitor buthionine sulfoximine (BSO) have shown that GSH is essential for the development of primary and lateral roots (LRs) [29,30]. One possible mechanism that could explain how GSH can affect primary and lateral root development is through the modulation of auxin transport by decreased expression of the PIN-FORMED (PIN) proteins [29,30]. The Arabidopsis genome has eight PIN genes (ATPIN1 to AtPIN8), five of which (ATPIN1, 2, 3, 4 and 7) comprise a subclade [31]. These PINs participate in plant development and in the integration of environmental stimuli [32,33,34,35,36]. Although these five PINs show different expression patterns, they partially overlap in the root meristem. PIN1 localization mainly occurs in an acropetal direction, toward the root apex, in vascular cells [31,34,37] and contribute to auxin movement to the root apical meristem (RAM). PIN2 is present basipetally, toward the base, in the membranes of epidermal cells and acropetally in the membranes of cortical cells [33,37], to establish a “reflux loop” in the root apex. PIN3 and PIN7 are localized without any pronounced polarity in the columella cells, and this localization could contribute to a lateral efflux from the root columella cells to mobilize auxin out of the root meristem [31,35,37]. PIN4 is detected in the plasma membrane of the quiescent center and in the surrounding cells, so it could also contribute to the reduction in auxin concentration in the RAM [34,37]. The proper functioning of these PIN proteins is essential for maintenance of the root apical meristem [37] and for establishment of the LR primordia [38].

PINs contribute to LR formation at the earliest stage (specification of LR founders in a subset of xylem pole pericycle cells). Specifically, PINs inhibit LR formation, based on the observation that a triple mutant *pin2*,*3*,*7* showed an increased LR density [39]. At his stage, the recurrent expression of the synthetic auxin-responsive promoter DR5, widely used for monitoring auxin concentrations and sensitivity [40], is observed in xylem pole pericycle cells. Similarly, the occurrence of the DR5 signaling peak is correlated with the sites of later LR initiation [41]. The next step of LR formation is LR initiation, which is characterized by the nuclear migration in founder cells correlated with DR5 reporter expression and, consequently, the first asymmetric cell division [42]. These features indicate that auxin signaling guides LR initiation. The specification of founder cells precedes cell division during primordium morphogenesis, as shown by the occurrence of DR5 activity in the pericycle cells in the *alf4-1* Arabidopsis mutant, despite the blockade of pericycle cell division in this mutant [43]. Following the different stages reveals that LR outgrowth, emergence, primordium patterning, and growth are mainly driven by signals originating from the LR itself [44]. However, LR outgrowth and emergence depend on shoot-driven auxin [45,46]. Given that LR outgrowth depends on shoot-derived auxin, experiments with roots with removed hypocotyls will help to identify the auxin response in LR primordia, independent of shoot-derived auxin.

In addition to polar auxin transport, local TRYPTOPHAN AMINOTRANSFERASE OF ARABIDOPSIS1 (TAA1)-dependent auxin biosynthesis is also crucial for RAM establishment [47] and development of LR [48]. However, whether TAA1-related auxin biosynthesis contributes to plant responses to GSH is unknown and requires further investigation.

The other potential mechanism of crosstalk between GSH and auxin is through modulation of auxin signaling. This crosstalk between GSH and auxin signaling is supported by the discovery of a key role for GSH in cell de-differentiation in alfalfa leaf protoplasts, wherein GSH acts synergistically with auxin [24]. However, further investigation is needed to determine whether this type of interaction also occurs in roots. The classical auxin signaling pathway involves AUXIN/INDOLE-3-ACETIC ACID (Aux/IAA) proteins, which are auxin-response inhibitors that bind to the AUXIN RESPONSIVE FACTOR (ARF) and, at low auxin concentrations, block the expression of auxin-responsive genes. Binding of auxin to TRANSPORT INHIBITOR RESPONSE1 (TIR1)/AUXIN-SIGNALING F-BOX (AFB) receptors stimulates the ubiquitination of Aux/IAA proteins and their proteasome-dependent degradation, resulting in the release of ARF and the induction of expression of auxin-responsive genes [49].

However, recent investigations of auxin responses have shown an involvement of alternative auxin signaling pathways that decouple the stability of Aux/IAA proteins and the transcription of auxin responsive genes [50,51]. These alternative pathways are based on specific ARFs that do not contain the conserved domain responsible for binding Aux/IAA proteins [52]; thus, the activity of these ARFs is independent of Aux/IAA stability. The presence of an alternative auxin signaling pathway emphasizes the importance of using different auxin-responsive markers that indicate both Aux/IAA stability and the transcription of auxin-responsive genes when studying the effect of different stimuli on auxin responses. The effects of GSH on the stabilization of Aux/IAA and on the transcription of classical auxin responsive genes are also unknown and require further investigation.

The aim of this study was to investigate the effect of GSH on auxin signaling in RAM and in the pericycle, which is the site of formation of the LR primordia. In RAM, we also studied the effect of GSH on the expression and localization of PIN proteins and on the expression of the TAA1 auxin biosynthesis protein. We estimated the cross-talk between GSH and auxin on LR initiation using isolated roots incubated in media with and without BSO and at different auxin concentrations to allow estimation of the signaling between externally applied auxin and internal GSH, while precluding any confounding results due to the delivery of auxin from the shoot.

## 2. Results

### 2.1. Gsh Controls Root Apical Meristem and Growth 0f Primary Root

**Figure 1:**
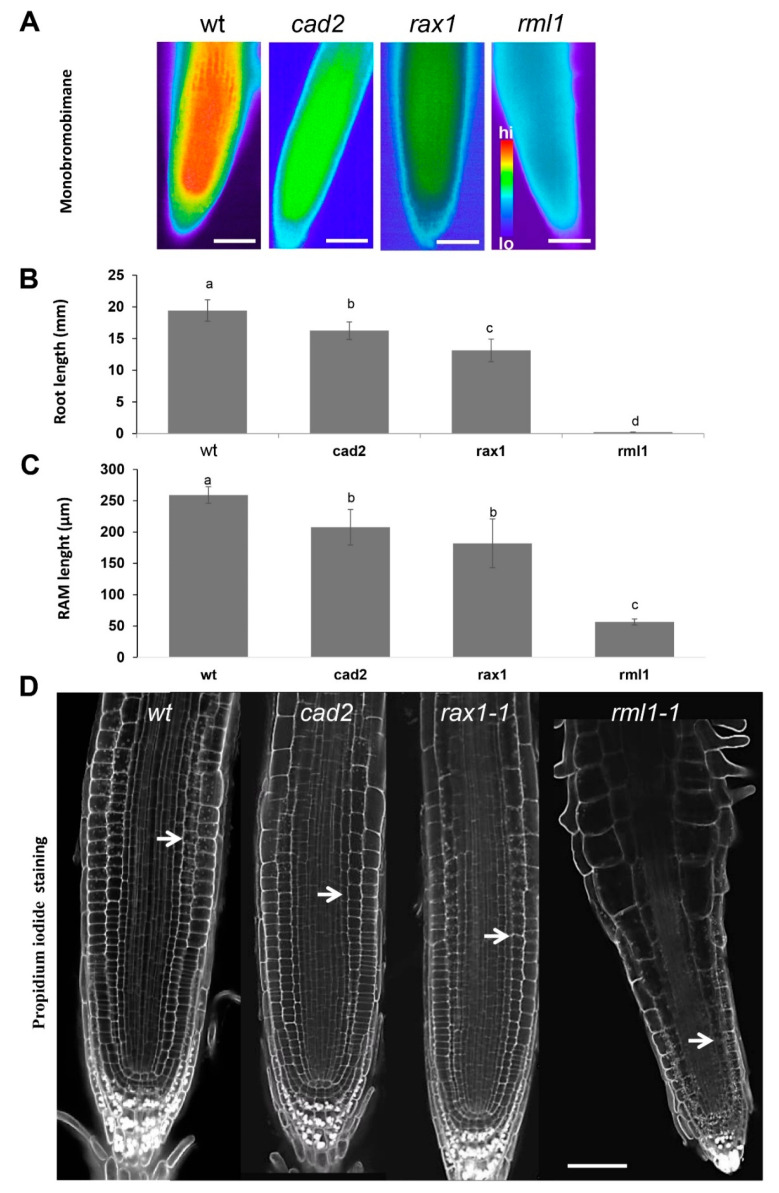
Root traits of wild type, *cad2*, *rax1*, and *rml1* mutants. (**A**) Semi-quantitative analysis of low molecular weight thiols using fluorescent in situ labeling with bromobimane. Scale bars represent 20 µm; (**B**) Root length after seven days of growth. Error bars represent the means ± SDs (*n* = 8); (**C**) Length of the root apical meristem at seven days after germination. Error bars represent the means ± SDs (*n* = 8). Different letters indicate a significant difference at *p* < 0.05. Fisher’s protected LSD test was used to compare the individual means; (**D**) root apical meristem (RAM) structure after propidium iodide (PI) staining. White arrow indicates the end of meristematic zone borders; Scale bars represent 100 µm.

We evaluated the effect of GSH on the root apical meristem (RAM) and on the growth of primary roots by analyzing the GSH-deficient mutants of the γ-EC synthase gene (*At4g23100*). This gene codes for the enzyme glutathione synthase1 (GSHS1) that performs the first step in GSH biosynthesis. These mutants include *cadmium-sensitive2-1* (*cad2*) [53], the *regulator of APX2 1-1* (*rax1-1)* [54], and *root-meristemless 1* (*rml1)* [21,55]. We first visualized GSH contents in these mutants semi-quantitatively by staining the thiols with the thiol-specific dye monobromobimane. This staining showed significantly lower GSH content in all mutants than in the wildtype controls, with the lowest GSH content in *rml1* (Figure 1A). These results are in agreement with previously published data, showing that *rax1-1*, *cad2*, and *rml1* mutants contain 32%, 28% [54], and 2.7% of the glutathione content of the wild-type [21].

In agreement with the monobromobimane assay results (Figure 1A) and previously measured GSH concentrations in wild type and mutant plants [21,54], the root growth of the *rml1* mutant was almost completely blocked and the root growth of *cad2* and *rax1* mutants was decreased by 80% and 65%, respectively, compared to the wild type (Figure 1B). Since the rate of root growth is affected by both cell division and cell elongation, the number of cortex cells was quantified in a cell file extending away from the apex, starting from the quiescent center (QC) and extending to the first cell that was twice as long as the shortest cell in the file. Consistent with the root growth data, the root apical meristem (RAM) was significantly decreased in the *cad2*, *rax1*, and especially in the *rml1* mutants, compared with the RAM in the wild type (Figure 1C,D).

**Figure 2:**
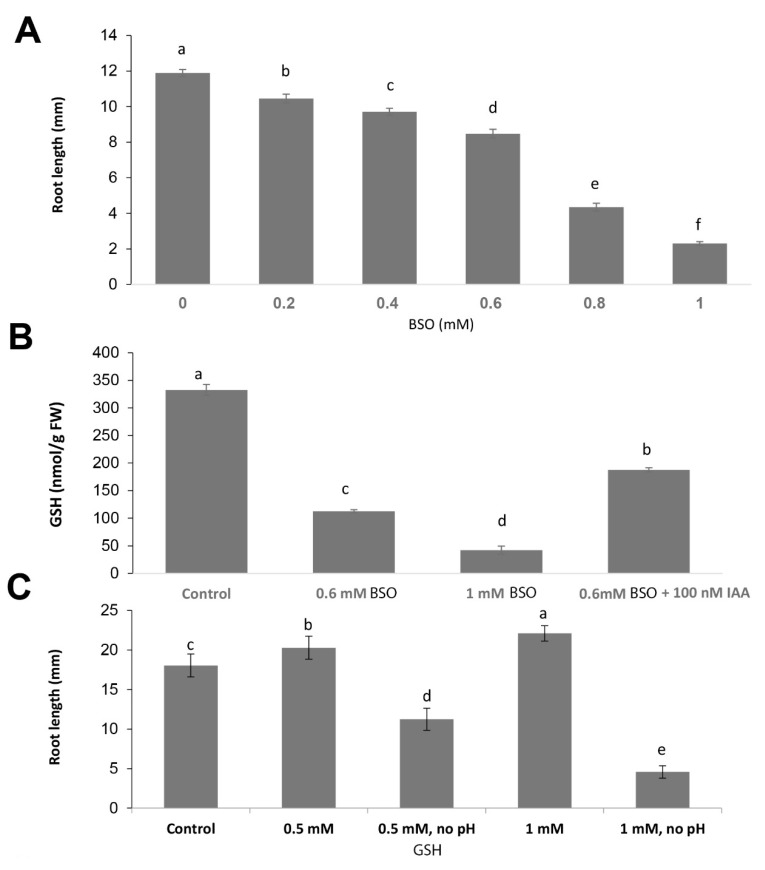

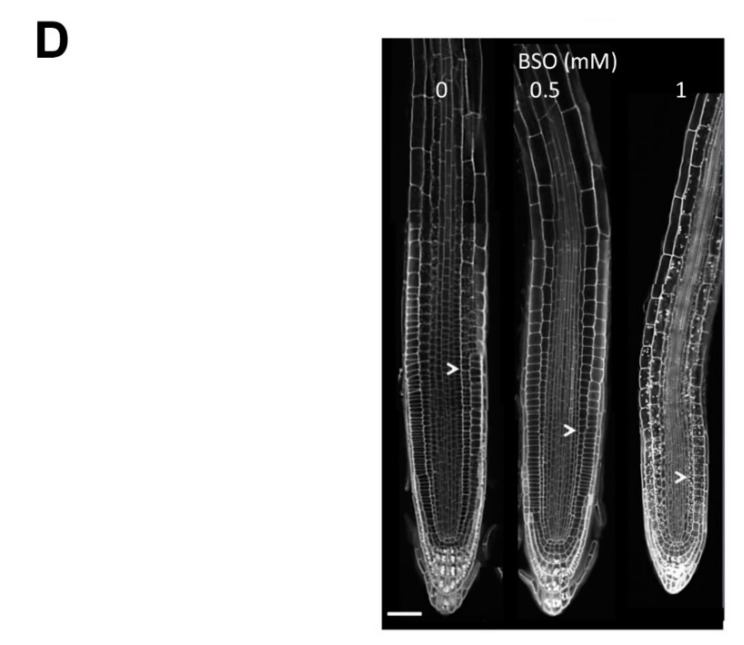
Pharmacological manipulation affecting tripeptide glutathione (GSH) contents and root growth. (**A**) Root length after five days of exposure to different concentration of L-buthionine-(R,S)-sulfoximine (BSO). Error bars represent the means ± SDs (*n* = 8–10); (**B**) GSH concentrations in roots of Arabidopsis seedlings after treatments with L-buthionine-(R,S)-sulfoximine (BSO) and BSO plus IAA. Error bars represent the means ± SEs (*n* = 3); (**C**) Root length after 5 days of exposure to different concentrations of GSH with and without adjustment of the pH of the growth medium. Error bars represent the means ± SDs (*n* = 10–16). Different letters indicate a significant difference at *p* < 0.05. Fisher’s protected LSD test was used to compare the individual means; (**D**) RAM structure after propidium iodide (PI) staining. White arrows show the end of meristematic zone borders. Scale bar −50 µm.

We further evaluated the effect of GSH on root growth in wildtype plants by changing the cellular GSH concentrations pharmacologically with exogenous application of BSO, a specific inhibitor of the γ-EC synthase, or GSH. Root length and GSH contents were significantly reduced in a dose-dependent manner after treatment with BSO (Figure 2A,B). Quantitative determinations revealed that the GSH contents had declined to 30% of the level in the wildtype at 0.6 mM BSO and to 10.2% following treatment with 1 mM BSO. By contrast, the addition of 100 nM IAA partially rescued this effect (Figure 2B). Following application of 1 mM BSO, the RAM length (determined as the number of cortex cells) contained only 7–8 cells per single lineage (Figure 2D).

External application of 0.5 mM and 1 mM GSH to the medium increased root growth by 15% and 20%, respectively (Figure 2C), supporting the stimulatory effect of GSH on root growth. However, this effect of GSH was only observed when the pH of the GSH stock solution was adjusted to match the pH of the growth medium. This is because GSH causes a significant acidification of the culture medium to pH 3.26 and 3.45 at 0.5 mM and 1 mM BSO, respectively, thereby resulting in a significant inhibition of root growth due to the acidic conditions in the medium (Figure 2C). This acidification of the medium by GSH is in agreement with previously published data [23].

### 2.2. Gsh Modulates Auxin Signaling and Auxin Biosynthesis in the Ram

**Figure 3:**
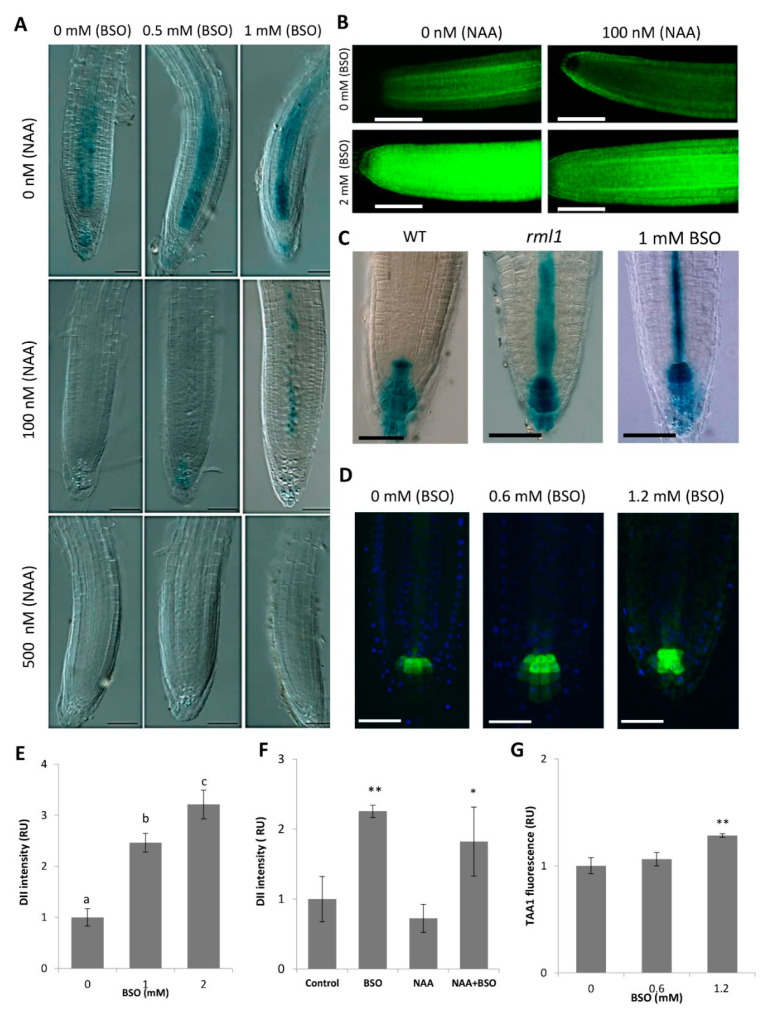
Effect of GSH level on auxin response markers and auxin biosynthesis gene expression. (**A**) BSO dose-dependently increases AXR3NT-GUS stability in response to auxin. Seven-day-old seedlings were treated for 12 h with different L-buthionine-(R,S)-sulfoximine (BSO) concentrations in the liquid medium and/or pre-treated in the control medium. Seedlings were subjected to heat shock by cultivation at 37 °C for 1.5 h, allowed to recover for the next 10 min, and then subjected to GUS assays for the next 1–1.5 h; (**B**) BSO increases DII::VENUS stability without and with NAA application; (**C**) DR5::GUS signal in wildtype seedlings, rml1 mutant seedlings, and seedlings treated with 1 mM BSO; (**D**) TAA1::GFP signal in RAM upon BSO treatment. Scale bar −50 µm. (**E**) DII::VENUS quantification in response to BSO concentrations. Error bars represent the means ± SDs (n = 6). Different letters indicate a significant difference at *p* < 0.05; (**F**) DII::VENUS quantification following BSO and NAA treatments (represented image in B). Error bars represent the means ± SEs (n = 6); (**G**) TAA1::GFP quantification in RAM (represented image from D). Error bars represent the means ± SDs (n = 8). Means of treatments with * and ** are significantly different from means of the control at *p* < 0.05 and *p* < 0.01, respectively. Fisher’s protected LSD test was used to compare the individual means.

Auxin is considered the principal hormonal candidate for the integration of the GSH effects on root growth and RAM activity [55,56]. We addressed the effect of GSH on the stabilization of Aux/IAA proteins, the inhibitors of auxin signaling using two reporter lines, HS::AXR3NT-GUS and DII-VENUS, that display auxin responses in relation to proteasome-dependent degradation [57,58]. Both these reporters are based on the DII domain of IAA proteins. The DII domain is necessary and sufficient for the interaction of IAA proteins with TIR1/AFB co-receptors and consequent IAA degradation. The degradation dynamics of these reporters provide an estimate of the input signal into the auxin signaling pathway. The activities of both markers were significantly higher at low GSH levels (i.e., after BSO treatment) (Figure 3A,B), suggesting a down regulation of auxin responses in these conditions. Consistently, the activities of the HS::AXR3NT-GUS reporter in the RAM and its activities upon treatment with exogenous auxin showed dose-dependent changes in response to BSO. Interestingly, 1 mM BSO partly blocked the degradation of the AXR3NT-GUS reporter in response to 0.1 µM IAA, suggesting that GSH enhances the sensitivity of auxin signaling (Figure 3A). Similar results were obtained with the DII::VENUS reporter, showing an induction of a higher VENUS signal in the presence of BSO (Figure 3E); however, this could not be completely offset by treatment with a low concentration (100 nM) of NAA (Figure 3,B,F).

**Figure 4:**
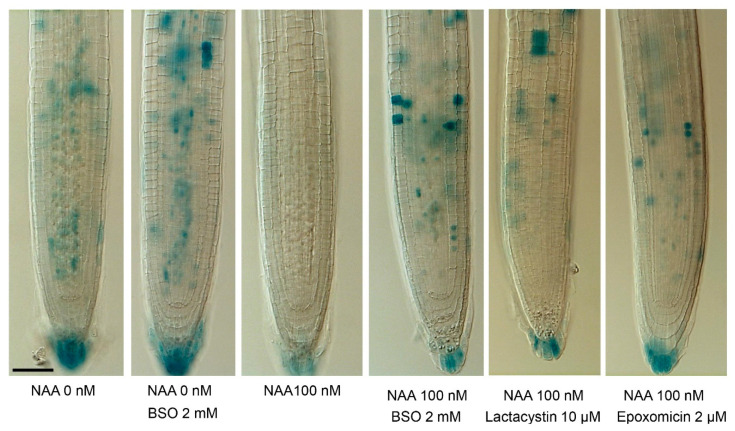
Effect of BSO, NAA, and proteasome inhibitors on the stability of AXR3NT-GUS. Four-day-old HS::AXR3NT-GUS seedlings were adapted in liquid medium and then treated for 12 h with BSO and 20 min with proteasome inhibitors. The seedlings were then treated for 1.5 h with 100 nM NAA and subjected to heat-shock at 37 °C for 1.5 h. GUS assays were performed for next 1.5 h. Scale bar −50 µm.

**Figure 5:**
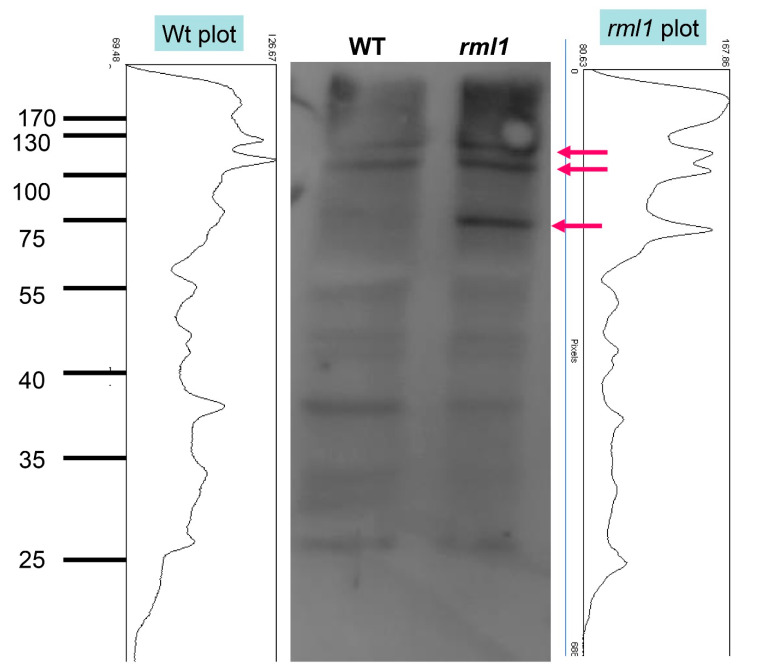
GSH level affects protein ubiquitination status. Protein was isolated from 4-day-old WT and homozygotic rml1 plants and subjected to immunoblotting with Ubi11 AS08 307 (Agrisera) antibody. Each lane contains 10 µg protein. Arrows show the accumulations of high MW ubiquitinated proteins in rml1 mutant.

Consistent with this finding, the effect of GSH deficiency on HS::AXR3NT-GUS marker expression was similar to that observed with well-known proteasome inhibitors like lactacystin and epoxomicin (Figure 4), thereby indirectly supporting the GSH effect on the proteasome-dependent degradation of Aux/IAA proteins. Western blot analysis of ubiquitinated proteins from 4-day-old wildtype and *rml1* mutant plants further confirmed an involvement of GSH status in protein polyubiquitination, which was higher in the GSH deficient *rml1* mutant than in the wildtype for proteins sized 70 kDa and larger (Figure 5). Taken together, these data suggest an important role of GSH in the regulation of the protein degradation process through proteasomes and may explain its impact on auxin-mediated responses in the RAM.

We estimated the effect of GSH on transcriptional regulation of auxin responsive genes using the artificial auxin response DR5::GUS marker activity, which reflects a combination of auxin response and abundance [59]. In the stationary phase, DR5 is expressed in the cells that maintain a consistently high auxin level through accumulation of shoot-derived auxin, as well as by internal auxin biosynthesis (through TAA1 activity). The activity of the DR5 promoter in the root tip did not differ significantly from the mock treatment under moderate BSO treatment (200–600 μM). However, the DR5 signal was significantly higher in the *rml1* mutant and upon exposure of the plants to a high BSO concentration (1 mM) (Figure 3C, Figure S1), suggesting that transcriptional auxin responses were enhanced under severe GSH deficiency. Interestingly, both cases of extreme GSH depletion (1 mM BSO and *rml1*) resulted in a strong signal of the auxin-related DR5 activity in columella cells and on its evident extension from the columella to the stele (Figure 3C). This increase in the DR5 signal was observed despite the increased stability of Aux/IAA proteins (Figure 3B) under GSH deficiency, thereby indicating the potential role of an alternative signaling pathway in the regulation of DR5, independent of Aux/IAA stability. One possible reason for this enhanced DR5 activity could be possible increased internal auxin biosynthesis. Investigations of TAA1 activity with the TAA1::GFP reporter line [47] showed that TAA1 activity was higher in columella cells after BSO treatment (Figure 3D,G), suggesting that GSH levels also have an impact on auxin biosynthesis.

### 2.3. GSH Level Affects PIN-Modulated Auxin Transport

**Figure 6:**
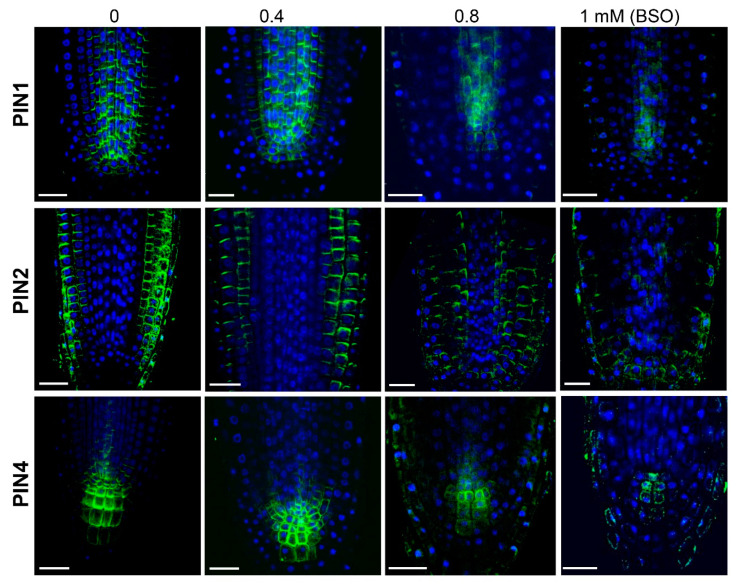
GSH level altered the PIN signal localization in the RAM. PIN1, PIN2, and PIN4 expression and localization in the RAM of Arabidopsis seedlings grown in a medium with different L-buthionine-(R,S)-sulfoximine (BSO) concentrations. Scale bar −20 µm.

Changes in the expression and polarity of the PIN proteins have been previously shown to alter the distribution of auxin within the root apical meristem, leading to changes in root morphogenesis [34,37]. In the *cad2* and *rax1* mutants, as well as in wildtype plants under moderate BSO treatments (up to 0.4 mM), PIN levels and localization appeared similar to those of untreated wild type plants, indicating that even a drop of 20% in the GSH level is not sufficient to interfere significantly with polar auxin transport in the RAM (Figure 6).

**Figure 7:**
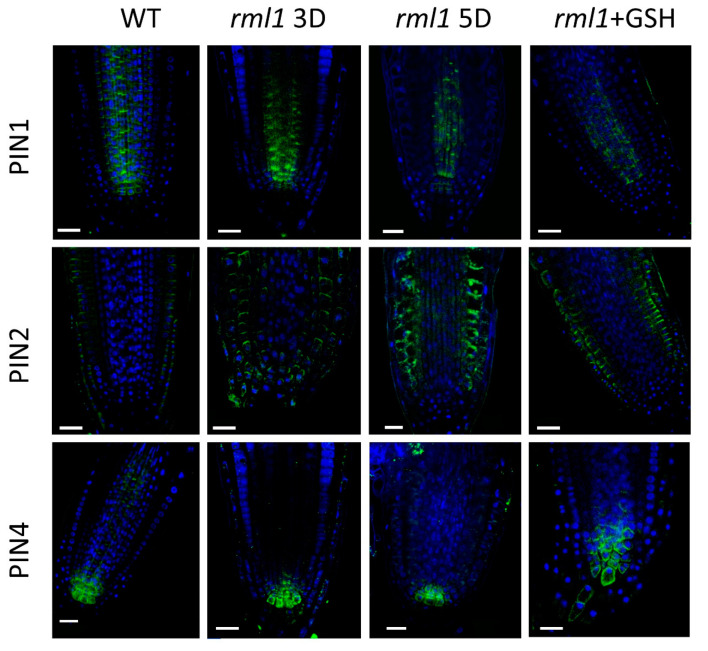
GSH level altered the PIN signal localization in the RAM. PIN1, PIN2, and PIN4 localization in rml1 mutant at 3 and 5 days after germination and rml1 mutants treated with 0.5 mM GSH. Scale bar −20 µm.

However, a more severe GSH depletion (in the *rml1* mutant and after exposure of the wildtype to 0.8 mM BSO) did impact the localization (polarity) and level of expression of the PIN1, PIN2, and PIN4 proteins and, hence, the overall functionality of the polar auxin transport machinery within the RAM (Figure 6 and Figure 7). Specifically, at 0.8 mM of BSO, PIN1 was localized at both the apical and basal parts, and a diffuse PIN1 signal was also visible in the cells. Treatment with 1 mM BSO further reduced PIN1 signaling and induced the re-localization of PIN1 in a basipetal direction opposite to its localization in the mock treatment. Similar to PIN1, visible down-regulation of expression and modulation of localization for PIN2 was observed in response to 0.8 mM BSO. The PIN2 signal in the plasma membrane of the epidermis and cortex decreased; moreover, ectopic PIN2 expression appeared in the endodermis, with a basal polar localization, and in the columella cells. A higher BSO concentration (1 mM) further decreased the PIN2 signal so that visible PIN2 expression was observed in the epidermis and columella cells, but not in the cortex cells. The PIN4 signal was strongly reduced at 0.8 mM BSO and maintained its typical membrane localization. A further increase in the BSO concentration (1 mM) induced vesicle formation in the cells, where PIN4 is typically expressed, indicating an involvement of GSH in PIN4 circulation. Analysis of the *rml1-1* mutant and the treatment of *rml1* mutant with 1 mM GSH (Figure 7) supported the observations with BSO-treated plants, as strong GSH deficiency reduced the expression of all PINs. The recovery of PIN expression pattern of *rml1* mutant with 1 mM GHS is in agreement with the recovery of root growth of *rml1* mutant seedling (Table S1).

The regulation of PIN circulation by redox signaling has been supported by an investigation showing the inhibition of PIN2 exocytosis by the reactive oxygen species, hydrogen peroxide [60]. Our recent investigation showed that GSH had a reverse response to that induced by hydrogen peroxide [61], which supported the regulation of PIN2 trafficking by the redox status of the cells. A complex feedback loop between redox signaling, auxin, and PIN expression and localization may therefore be affecting the RAM, as redox homeostasis affects PIN expression and localization, while PINs modulate the auxin gradient in the RAM [30] and change auxin concentrations, whereas the modulation of auxin signaling in turns affects PIN expression [62].

### 2.4. GSH Enhances Auxin Signaling in Isolated Root Segments

**Figure 8:**
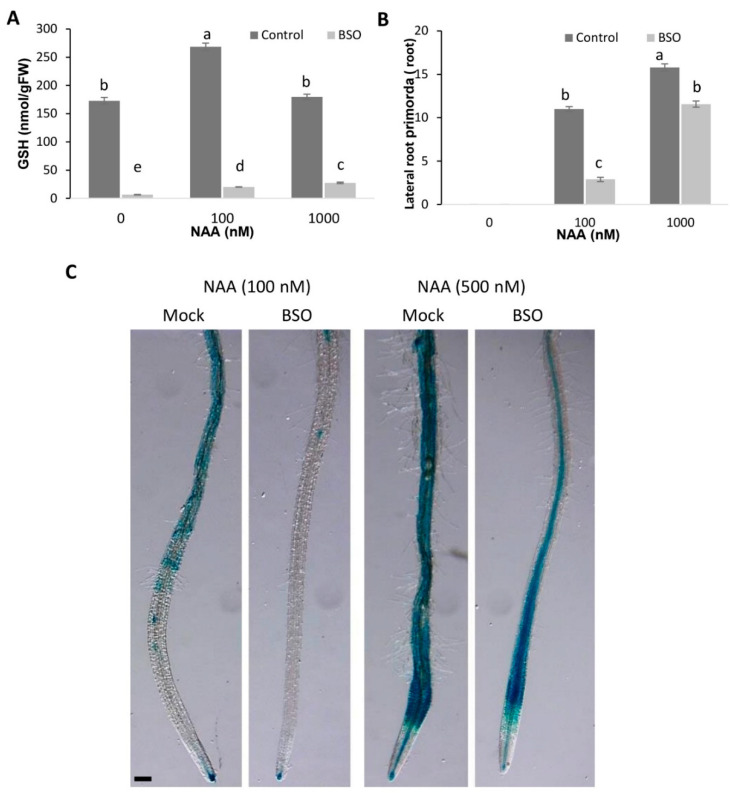
GSH affected the induction of lateral root primordia and the DR5 signal in isolated roots. (**A**) The effect of 1 mM L-buthionine-(R,S)-sulfoximine (BSO) and different NAA concentrations on GSH concentrations in isolated roots. Error bars represent the means ± SEs (*n* = 3); (**B**) BSO inhibited de novo formation of the lateral root primordia. Error bars represent the means ± SEs (*n* = 10). Different letters indicate a significant difference at *p* < 0.05. Fisher’s protected LSD test was used to compare the individual means; (**C**) BSO inhibited the auxin response, determined as DR5 activity, after NAA treatments. Scale bar −100 µm.

Lateral root formation depends on auxin transport from the shoot [63]. We estimated the direct effect of auxin signaling by excluding the involvement of shoot-derived auxin on LR formation using isolated roots. The segments were incubated in medium with or without BSO and with different auxin concentrations. We ensured that auxin signaling occurred independently of auxin effects on GSH concentration by measuring the effect of NAA on GSH concentrations in roots incubated with or without BSO. The application of 1 mM of BSO strongly reduced the GSH concentration in the isolated roots to a very low level (Figure 8A).

We found that treatment with 0.1 µM NAA increased the GSH concentration in the roots, either not treated or treated with BSO; moreover, increasing the NAA concentration to 1 µM further increased the GSH concentration in BSO-treated roots. Taking into account that relatively low GSH concentration (20%) is sufficient to maintain many root functions, we assume that this auxin-induced increase in GSH concentration can partly contribute to the recovery of the auxin response in BSO-treated roots.

**Figure 9:**
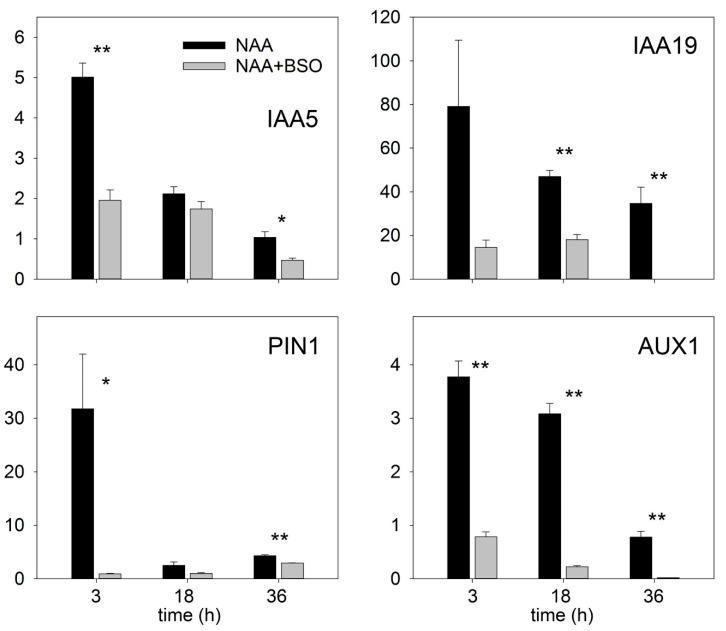
GSH is important for the auxin-induced response. GSH regulated the transcription of classical auxin-responsive genes according to real-time qPCR assays. Isolated roots from 7-day-old plants were pre-treated with 0 and 2 mM L-buthionine-(R,S)-sulfoximine (BSO) for 12 h and then treated with 100 nM NAA for the indicated times. Error bars represent the means ± SEs (*n* = 3). Means of treatments marked with * and ** are significantly different from means of the control at *p* < 0.05 and *p* < 0.01, respectively. Student’s *t*-test was used.

The transcriptional auxin response of isolated root segments was observed using the DR5 auxin marker after incubation of the roots with a low auxin concentration (0.1 µM). At this auxin concentration, GSH depletion of the roots by BSO treatment strongly inhibited transcriptional auxin signaling, as indicated by the low DR5::GUS signal in the roots. However, a higher auxin concentration (0.5 µM) can compromise transcriptional auxin signaling, suggesting that a strongly reduced GSH level in plants cannot block auxin signaling. However, at both tested auxin concentrations (0.1 and 0.5 µM), the DR5 signal was weaker in GSH-depleted plants, suggesting a role for GSH in the enhancement of auxin signaling (Figure 8C). We confirmed the effect of GSH on the transcriptional auxin response by investigating the expression of other auxin response markers, such as IAA5, IAA19, PIN1, AUX1, and found that these were up-regulated by auxin only if the GSH pool was not depleted (Figure 9). This finding supported a key role for GSH in stimulating the transcriptional induction of auxin-responsive genes. The presence of GSH was also important for the regulation of CycB1::GUS activities (Figure S2) and, ultimately, LR formation.

Importantly, higher NAA concentrations (0.5 µM) could partially diminish the effect of GSH deficiency after BSO treatments by rescuing DR5 expression and increasing the frequency of induction of de novo LR primordia (Figure 8B,C). However, higher NAA concentration failed to induce cell division in the *rml1* mutant. This indicates that GSH not only increased the sensitivity of roots to auxin, but that a minimal GSH concentration is required (and cannot be compensated by auxin) for LR development.

Collectively, our results showed that GSH enhances auxin signaling because GSH stimulates Aux/IAA degradation, the expression of classical auxin responsive genes, the progression of cell division, and LR formation.

## 3. Discussion

GSH is considered one of the most important cellular redox buffers and a signaling molecular involved in plant responses to different abiotic and biotic stresses. As a molecular signal, GSH interacts with auxin action responses to allow plant adaptation to environmental stimuli. However, the type of interaction between GSH and auxin differs in the RAM and in the pericycle, the site of formation of LR primordia. In the RAM, low GSH stabilized Aux/IAA proteins; but increased the DR5 signal in QC and adjusted cells. By contrast, in the pericycle, low GSH stabilized Aux/IAA and inhibited DR5 expression.

### 3.1. Glutathione Increases Auxin Sensitivity by Interfering with Ubiquitin-Mediated Proteolysis

The results reported here showed that GSH is a significant enhancer of auxin signaling. The evidence for GSH enhancement of auxin signaling is our finding that GSH deficiency increased the stability of Aux/IAA proteins, modulated the total ubiquitination of proteins, inhibited the expression of classical auxin responsive genes and a DR5 auxin response marker in the pericycle, and inhibited the formation of LR primordia.

The key to auxin signaling is the ubiquitin-proteasome–dependent degradation of Aux/IAA proteins, which are repressors of auxin signaling. This degradation occurs after auxin binds to TIR1, the F-box protein subunit of an ubiquitin protein ligase (E3) called SCF^TIR1^, as this triggers consequent Aux/IAA ubiquitination [64]. Degradation of Aux/IAA releases ARF binding to auxin-responsive elements in the promoter sequences of auxin-responsive genes that induce the expression of auxin responsive genes, and this can be followed by the expression of a DR5 auxin-responsive marker.

In our study, the stabilization of both the AXR3NT-GUS and DII-VENUS Aux/IAA markers in GSH-deficient plants under control conditions and following exposure of Arabidopsis seedlings to auxin (0.1 µM) strongly indicates the inhibition of auxin signaling. However, the fact that a higher auxin concentration (0.5–1 µM) induced the degradation of Aux/IAA under GSH deficiency, as well as affecting other auxin-related markers (e.g., the induction of DR5 expression and de novo formation of LR primordia) (Figure 8B,C), indicated that a higher auxin concentration can compromise GSH deficiency. Therefore, maintaining of GSH concentration in optimal range in plants has stimulating character on auxin responses. However, the minimal concentration of GSH is required for auxin response because auxin cannot rescue cell division in the low-GSH content *rml1* mutant. 

One reason why the GSH deficiency can be offset by a higher auxin concentration is that auxin can increase GSH biosynthesis in GSH-deficient plants. Indeed, in our study, IAA exposure of BSO-treated plants partially restored the level of GSH, indicating that the rescue effect of IAA at high concentration can be related, at least partly, to an increased GSH concentration in IAA-treated seedlings. In agreement with this observation, a stimulatory effect of auxin on GSH concentration has been reported in experiments with isolated pea internode segments and *Avena* coleoptile sections [65].

We found a strong modulation of total protein ubiquitination in roots of the low-GSH content *rml1* mutant, indicating that GSH does not affect Aux/IAA proteins specifically, but instead interferes with the ubiquitination machinery in plants. This finding rationalizes why GSH would interact not only with auxin, but also with other hormones, including jasmonate (JA), gibberellin, strigolactone, ethylene, abscisic acid (ABA), and salicylic acid (SA), where ubiquitin-proteasome-dependent degradation is involved in the regulation of gene expression [66,67]. Indeed, transcriptomic analysis in experiments where GSH level was modulated in plants showed that ABA, JA, ethylene, and SA signaling are also involved in cross-talk with GSH signaling [68,69].

The mechanism responsible for ubiquitination by GSH in plants is not known. Examples from studies on human cells showed the potential role of de-ubiquitinases in the modulation of protein ubiquitination; these enzymes can be reversibly inhibited by ROS due to the oxidation of their catalytic cysteine [70]. Our observation that the treatments with proteasome inhibitors induced a similar phenotype to that induced by BSO, which inhibits the degradation of AXR::NY::GUS, further supports the hypothesis that a deficient level of GSH interferes with proteolysis in root cells and affects the stabilization of Aux/IAA proteins that ultimately inhibits the expression of auxin-responsive genes. In agreement with this hypothesis, the expression of auxin-responsive genes and the DR5 auxin marker was inhibited in the elongation and differentiation zones of roots with low GSH content (Figure 8C and Figure 9). However, the DR5 signal was increased in columella, quiescent center (QC), and stele cells adjusted to the QC, assuming that tissue-specific interactions are possible in cross-talk between GSH and auxin.

### 3.2. Cross-Talk Between Glutathione and Auxins in the Apical Meristem

Within the RAM, the QC cells are in a highly oxidized environment, which is characterized by a low ratio of GSH to GSSG and of ASC to DHA compared with their adjacent, rapidly dividing cells [71]. GSH contributes positively to auxin signaling and auxin application shifts the GSH/GS-SG ratio in favor of the reduced form; however, the oxidized environment in the RAM is co-localized with a DR5 auxin maximum. This inconsistency reflects the fact that the intensity of the DR5 signal depends not only on the sensitivity of the auxin signaling machinery, but also the auxin concentration, which in turn depends on auxin biosynthesis and auxin transport. Polar auxin transport from the shoot to the roots has long been considered a main contributor of auxin in the RAM [72]. However, not all PINs are responsible for increases in auxin concentration in the RAM due to auxin transport from shoot to roots. On the contrary, PINs can also contribute to reductions in the auxin concentration in the RAM. For example, a mutation in *PIN4* increases the DR5 signal in the roots, assuming that PIN4 contributes to the sink-driven auxin gradient in the Arabidopsis root tip [34]. PIN2 can also decrease the auxin concentration in columella cells [73], because PIN2 is important for basipetal auxin transport (from the tip) [37]. The effect of PINs on auxin distribution and the previously identified upregulation of PIN proteins by auxin [62] contribute to positive feedback of the auxin transport machinery, thereby supporting the canalization hypothesis [74]. However, under GSH deficiency, despite a higher DR5 signal (Figure 3C) and upregulation of the TAA1 auxin biosynthesis gene (Figure 3D,G), the level of PIN proteins decreased, indicating that glutathione deficiency has a more dominant effect on PIN expression than is observed with auxin.

Investigations of weakly ethylene-insensitive mutants determined that local auxin production, in addition to polar auxin transport, is critical for the generation of robust auxin gradients and the final root pattern formation [47]. Thus, increases in the DR5 signal in QC and columella cells in plants under controlled conditions might be related to the localization of auxin biosynthesis and the contribution of PIN-modulated polar auxin transport, despite a potential reduction in the sensitivity of the signaling machinery due to oxidized environment in the RAM. Experiments with an exogenously supplied ASC precursor (L-galactono-y-lactone) and the oxidized form of ascorbic acid (DHA) showed increases and decreases, respectively, of the DR5 signal [75], supporting the hypothesis that oxidized conditions decrease auxin sensitivity. However, our experiment in which GSH concentrations were strongly reduced in the roots of an *rml1* mutant and with a high concentration of BSO showed that the DR5 signal increased, rather than decreased, in the columella cells, the QC, and the adjusted stele cells.

This increase in the DR5 signal in the GSH-deficient mutant might occur because of the modulation of activity of auxin biosynthesis in the RAM and modulation of polar auxin transport. Moreover, the ultimate triggers generated by GSH deficiency and by DHA might differ and may not be related only to the redox potential in the cells. Our studies of PIN expression and expression of TAA1 gene, which is a major contributor to auxin production in the RAM, showed that increased auxin biosynthesis through TAA1 might be the main mechanism responsible for the increased DR5 signal in the RAM. Evaluation of the contribution of PIN-modulated auxin transport is difficult because the various PINs contribute differently to auxin accumulation in the RAM. However, our finding that PIN2 and PIN4 were strongly downregulated by GSH deficiency indicates a potential contribution of reduced PIN2 and PIN4 expression to the increased DR5 signal in the QC and surrounding cells, because of roles of PIN2 and PIN4 in the reduction of the auxin signal in the RAM [34,73]. Previous investigations also showed that glutathione deficiency decreases PIN3 expression [29]. High co-regulation of PIN3 and PIN7 expression in Arabidopsis [31], coupled with the fact that these proteins contribute to lateral auxin redistribution [76], support their potential contribution to modulation of the DR5 signal in root meristem.

The discrepancy between Aux/IAA stabilization and DR5 upregulation might be due to the modulation of auxin concentration induced by biosynthesis and by polar auxin transport; however, it might also be related to alternative auxin signaling pathways that regulate auxin-responsive genes in parallel with the canonical TIR1/Aux/IAA-dependent pathway. The alternative pathway involves the auxin-mediated C-terminal cleavage of transmembrane kinase1 (TMK1), which contributes to the regulation of auxin responsive genes in the hypocotyl, LRs, and RAM [77,78,79,80]. Moreover, experiments with high spatiotemporal resolution have shown that root growth inhibition was dependent on TIR1/AFB signaling and Aux/IAA stability, but independent of transcriptional regulation [51]. This further supports a potential uncoupling between Aux/IAA stability and the expression of auxin-responsive genes.

Increases in the DR5 signal in the RAM under GSH deficiency indicate that inhibition of root growth does not occur due to a reduced transcriptional auxin response in the QC and surrounding cells. Thus, we assume that root growth inhibition was directly related to the more oxidized environments that stop cell proliferation, thereby supporting a direct role of GSH in cell proliferation [25,81,82]. Despite the positive role of GSH in cell proliferation, which has been well established in several investigations, GSH had a negative effect on root growth [20,29,83]. By contrast, other studies have shown that GSH stimulated root growth, especially during root growth recovery following the simultaneous application of heavy metals [84]. This discrepancy might be related to an indirect effect of GSH on the pH of the nutrient medium.

GSH is a weak acid that exerts a significant effect on the pH in the medium if used at millimolar concentrations. Indeed, in our study with Arabidopsis, we found that the effect of GSH on root growth strongly depended on whether the pH was controlled in the medium. GSH stimulated root growth under conditions where pH was maintained at the optimal range, whereas GSH inhibited root growth when the pH was not controlled.

### 3.3. Cross-Talk between Glutathione and Auxins by the Origination of Lateral Roots

The isolated root is a much more suitable system for studying auxin responses than roots of intact plants or the RAM, because auxin transport from the shoot, which contributes significantly to LR formation, is excluded in that model system. Using isolated roots, we found that GSH increases auxin sensitivity, as evident from Aux/IAA stability, the DR5 auxin response, and the initiation of LR primordia. The inhibition of LR formation in GSH-deficient plants is in agreement with previous investigations showing defects in LR formation in GSH-deficient mutants and BSO-treated plants, and with a key role of GSH in cell cycle progression in the G1 phase [21,22]. During cell cycle progression, GSH is massively recruited into the nucleus in dividing pericycle cells of the LRs [85]. This nuclear GSH recruitment promotes oxidation in the cytosol and enhances GSH biosynthesis [85].

In Arabidopsis, the LR is formed from founder cells that are induced early and occur at 3 to 8 mm from the root tip [86]. De novo lateral induction occurs in the cells located between the founder cells required for de novo cell de-differentiation. We excluded effects of GSH/BSO on founder cell establishment by observing LR initiation in the mature zone, where the number of founder cells is already determined. Our observation that low GSH inhibited the auxin-induced activation of LR formation suggests that GSH is required for cell transition from G1 phase as well as for de-differentiation or processes related to the re-specification of root pericycle cells into LR founder cells. This conclusion concurred with our previously published data with single cells of *Medicago sativa*, where GSH-induced cells underwent de-differentiation only in the presence of exogenous auxin [24]. Thus, GSH is involved in the auxin-induced G0-G1 transition in the pericycle cells.

## 4. Outlook

Cross-talk between ROS and auxin defines the plant growth response under environmental stresses [87]. The stress-induced morphogenic responses (SIMRs) are considered part of a general acclimation strategy and are characterized by the inhibition of root elongation and the enhanced formation of LRs [1]. ROS can oxidize GSH; however, ROS can also increase GSH concentrations due to the activation of GSH biosynthesis [88,89,90,91,92]. The cell proliferation activity of GSH and its response to ROS assumes an involvement of GSH in SIMRs. The inhibition of root elongation and enhanced formation of LRs might be related to the differential regulation of GSH concentration in the roots, and this requires further investigation.

## 5. Materials and Methods

### 5.1. Plant Material and Treatments

The genotypes used were wildtype *Arabidopsis thaliana* (L.) Heyhn. and the GSH synthesis mutants allelic for the enzyme glutamate-cysteine ligase (γ-ECS; At4g23100, EC 6.3.2.2): *rml1* [21,55], *rax1-1* [54] and *cad2-1* [53]. The HS::AXR3NT-GUS line [57] was kindly provided by Mark Estelle. DII::VENUS, TAA1::GFP, and CycB1::GUS by authors [47,58,93]. The *DR5::GUS* construct [59] was introduced in different mutant backgrounds through genetic crossing.

Seeds were surface-sterilized and sown on square Petri dishes in half-strength Murashige and Skoog (MS) medium [94], containing 1% sucrose and 1.1% agar (Roth, 4508.1), pH 5.6. Seeds were kept at room temperature for 4 h to allow water uptake, then transferred to a 4 °C for 12–14 h, and finally grown vertically in plates at 22 °C in a 16h/8h day night cycle under white light with 80 µmol s^-1^ m^-2^ intensity. Plates were scanned with an Adobe 950 scanner, and root lengths were measured using Image J software (https://imagej.net/Fiji). All auxin treatments of the seedlings were conducted in liquid medium. Seedlings were pre-cultured for at least 6 h in half-strength MS medium supplemented with 1% sucrose. Afterward, the only difference between cultures was the addition of one of the chemicals, as explained below.

For pharmacological treatments, GSH and its biosynthesis inhibitor L-buthionine-(R,S)-sulfoximine (BSO) were dissolved in water and the pH was adjusted to pH 5.6. BSO was added as diastereoisomer (Sigma-Aldrich, St. Louis, MO, USA). All stock solutions were filter-sterilized through a 0.22 µm filter (Roth) and added to the medium after it had cooled to 40 °C. Glutathione (GSH), indole-acetic acid (IAA), naphthyl acetic acid (NAA), and MG132 were obtained from Sigma (Sigma-Aldrich, St. Louis, MO, USA, C-2211). Epoxomicin (Cat. Number I-112) and lactacystin (Cat. Number I-112) were obtained from Boston Biochem, http://www.bostonbiochem.com/. Epoxomicin, IAA, NAA, and MG132 were dissolved in DMSO at 10 mM and lactacystin at 2 mM. All experiments present at least 3 independent biological replicates with at least 10 plants in each group.

### 5.2. Isolated Root Experiments

For isolated root experiments, the primary roots from 7-day-old seedlings were excised under a stereo-microscope and placed in a 40 mm Petri plate containing half-strength MS medium. The isolated roots were then pre-cultured for 6 h in the presence of BSO to reduce the endogenous GSH level. They were then treated with exogenous auxin (as indicated for the different experiments), followed by GUS assays, RNA isolation, or GSH determination.

### 5.3. Whole-mount In Situ Immunolocalization

Immunolocalization in Arabidopsis plants was performed according to a whole-mounting in situ protocol described in [95]. Affinity purified primary anti-PIN1 (mouse), anti-PIN2 (guinea pig), and anti-PIN4 (rabbit) antibodies were diluted 1:40, 1:400, and 1:400, respectively. The Alexa-488 anti-mouse (for PIN1), Alexa-488 anti-guinea pig (for PIN2), and Alexa 555-conjugated anti-rabbit (for PIN4) secondary antibodies were diluted 1:400.

### 5.4. GUS Assay

DR5::GUS and HS::AXR3NT-GUS reporter activity was determined using a standard GUS histochemical staining procedure. HS::AXR3NT-GUS seedlings were treated with BSO for 6 h or with proteasome inhibitors for 2 h. NAA was subsequently added for the next 90 min at the indicated concentrations, and the seedlings were heat-shocked at 37 °C for 90 min. The seedlings were then soaked in GUS staining solution (1 mg mL^−1^ X-GLUC in sodium phosphate buffer containing 1 mM EDTA and 2 mM ferric cyanide) at pH 7.0, incubated for 1–8 h at 37 °C, and then immersed in methanol. After 15 min, water was added gradually until a final methanol concentration of 15% was obtained. The samples were washed with water and subsequently cleared in 8:3:1 (*v*/*v*) chloral hydrate:distilled water:glycerol for 10 min. Samples were visualized using Nomarski optics on an Axioplan 2 (Zeiss, Oberkochen, Germany) microscope, using the Axiovision 3.1 (Zeiss, Jena, Germany) software for image processing.

### 5.5. Quantitative Real-Time PCR

Roots were collected at the indicated time points and transferred immediately to liquid nitrogen. The TRIzol reagent (Invitrogen, Carlsbad, CA, USA) was used to isolate total RNA. First-strand cDNA was synthesized with oligo(dT)_18_ primers using the First Strand cDNA Synthesis Kit (Fermentas, Waltham, MA, USA). Quantitative real-time PCR was carried out using the POWER SYBR GREEN PCR master mix (Roche, Basel, Switzerland) and gene-specific primers (Table S2) in a Light Cycler 480 Real-Time PCR System (Roche). Gene expression data were analyzed statistically according to Livak et al. [96]. Expression levels were normalized to actin (*ACT2*) expression levels.

### 5.6. HPLC Determination of GSH

The GSH concentration in the growth medium was determined by mixing the medium with an equal amount of ice-cold 3% (*w*/*v*) m-phosphoric acid and snap-freezing in liquid nitrogen. Tissue samples were weiged and ground in 3% (*w*/*v*) m-phosphoric acid on ice, followed by centrifugation at 50,000× *g* for 15 min at 4 °C. GSH determinations were carried out by reverse-phase HPLC on Polaris 3 reverse-phase C-18 column (3 µm particle diameter, Chromsep SS 100 × 4.6 mm, Varian Europe, Middelburg, the Netherlands) held at a constant temperature of 40 °C in a column oven (CTO-10AVP, Shimadzu, The Netherlands). The flow was driven by an isocratic pump (LC-10ADVP, Shimadzu, The Netherlands). The mobile phase consisted of 2 mM KCl, set at pH 2.5 by dropwise addition of concentrated o-phosphoric acid. The volume flow rate was set at 0.8 mol min^−1^. Oxygen was removed from the mobile phase by passing the flow through a degasser (DGU-14A, Shimadzu, The Netherlands). Injections were performed via an autosampling unit (SIL-10ADVP, Shimadzu, The Netherlands). Detection was via diode array (SPD-M10AVP, Shimadzu, The Netherlands), measuring between 196 nm and 250 nm, and set in tandem with a homemade amperometric detection system (glassy carbon working electrode, calomel reference electrode, reference potential 1000 mV). The latter was kept at a constant temperature within the column oven. The electrochemical detector was connected to a personal computer via an SS420 board (Shimadzu, The Netherlands). Chromatograms were analyzed with the ClassVP software package (Shimadzu HPLC Class VP 612 SP5, Shimadzu, The Netherlands). The whole system was controlled via the personal computer, in conjunction with a separate system controlling unit (SCL-10AVP, Shimadzu, The Netherlands).

Total GSH (GSH + oxidised glutathione [GSSG]) was determined by reducing 20 µL of each sample with 10 µL of a 200 mM dithiothreitol + 400 mM Tris solution (pH 6, checked in random samples). After a 20 min incubation at room temperature, the samples were acidified by addition of 10 µL 3% m-phosphoric acid and kept at 4 °C until injection. The GSSG concentration was calculated as half of the difference between the reduced GSH and total glutathione concentration.

### 5.7. Root Structure

Propidium iodide (PI) staining was conducted according to [97], with major modifications. Seedlings were fixed in 2% formaldehyde (freshly prepared from paraformaldehyde powder) in microtubule stabilization buffer (MTSB: 25 mM PIPES, 2.5 mM EGTA, 2.5 mM MgSO_4_, adjusted to pH 6.9 with KOH) for 30 min and washed three times with distilled H_2_O. Seedlings were incubated for 20 min in 100% methanol at 60 °C, with the subsequent gradual addition of water, until a final methanol concentration of 15% was obtained. Seedlings were washed once in water and incubated for 20 min in 1% periodic acid. After washing once again with water, the seedlings were incubated for 20 min in 100 mM Na_2_SO_3_, 0.15 M HCl, 0.2 mg/ml PI (pH 1.5) and washed with H_2_O. The seedlings were mounted in a chloral hydrate solution on microscopic slides with 150 µm spacers to keep the 3D structure intact. After staining, the seedlings were observed with a confocal laser scanning microscope (LSM Duo-Live, Zeiss, Jena, Germany) equipped with a He-Ne laser (561 nm excitation and 650–750 nm emission).

### 5.8. Western Blot and Ubiquitination Assay

Homozygotic *rml1* plants were selected at 3–4 days after germination. Proteins were isolated from selected seedlings and separated on polyacrylamide gel under standard conditions, except that the pH of the separation gel was adjusted to 9.1 [98]. The proteins were transferred to PDVF membranes, blocked with 2% milk, and incubated with primary antibodies overnight on a glass surface at 4°C under Saran-Wrap film. Membranes were given three 5 min washes in TBST buffer (1.211 g Tris, 8.766 g NaCl, 0.5 mL Tween-20, and 0.1 g NaN_3_ in 1 L ddH_2_O), incubated with the secondary antibody at room temperature for 45 min, and then examined using the Pierce chemiluminescence detection system. The following antibodies and dilutions were used in this study: Ubi11 (Agrisera, Vännäs, Sweden, AS08307, diluted 1:1500), anti-Ubi (K48) (1:2000, Millipore, Burlington, MA, USA) and anti-Ubi (K63) (1:2000, Millipore).

### 5.9. Thiol Localizations

The GSH-sensitive probe monobromobimane (M-20381, Invitrogen) was used for intracellular superoxide and thiol determinations. Dye was dissolved in DMSO at a concentration of 5 mM. Seedlings were cultured in liquid growth medium for at least 4–6 h for adaptation to the medium. The dye was then added to the medium at a final concentration of 10 µM and the seedlings were incubated in the dark for the next 10–20 min. Thereafter, the seedlings were carefully mounted in the original medium on microscope slides containing double spacers to prevent damage to the seedlings. To avoid dye bleaching, the focus was adjusted under a low-intensity halogen-lamp light. Illumination with fluorescent light (HBO-lamp) was restricted to a duration of 100 milliseconds. Slides were observed with a Zeiss Axiovert 200M inverted fluorescence microscope with a 20×/1- 1.6 NA objective lens. All images were acquired with a CCD camera (Sensys; Roper Scientific, http://www.roperscientific.com). The imaging setup was automatically controlled by MetaMorph/MetaFluor software (Universal Imaging, http://www.moleculardevices.com).

In vivo analysis of the fluorescence signal was done with a Zeiss Stemi SV11 APO stereomicroscope equipped with an HBO fluorescence microscopy lamp with a GFP filter set (488 nm excitation and 530–550 nm emission). For high-resolution images, plants containing fluorescent markers were fixed with 4% formaldehyde and mounted in Prolong Gold anti-fade reagent containing DAPI (Molecular Probes). Fluorescent proteins were analyzed with a Zeiss LSM 5 *DUO* scanning microscope. We monitored GFP and DAPI fluorescence using multi-tracking in frame mode. GFP (DII::VEBUS and TAA1::GFP) and Alexa Fluor 488 (for PIN1 and PIN2) were excited using the 488 nm laser line in conjunction with a 505–530 band-pass filter. Alexa Fluor 555 was excited with a helium-neon 543 nm laser (HeNe leser) in combination with a 575-long-pass filter. DAPI was excited with the 405 nm laser lines and collected using a 420–480 nm band-pass filter.

Data were statistically analyzed by analysis of variance (one-way ANOVA). When significant treatment effects were identified by ANOVA, Fisher’s protected LSD test was used to compare the individual means (using Statistica for Windows, version 13). Student’s *t*-test was used when only two treatments were compared.

## Supplementary Materials

Supplementary materials can be found at https://www.mdpi.com/2218-273X/10/11/1550/s1, Figure S1: The effect of BSO concentrations on the DR5::GUS signal in Arabidopsis roots; Figure S2: BSO prevents NAA-induced activation of the cell division in the distal pericycle zone. Five-day-old roots were treated with 100 nM NAA or 100 nM NAA+ 2 mM BSO for 36 hours. Scale bar is 100 µm. CyCB1::GUS was detected; Table S1: Root length in *rml1* mutant and *rml1* mutants treated with 1 mM GSH. Three-day-old *rml1* seedlings were treated with 1 mM GSH or without (mock treated) during 3 days. Means ± SDs are presented; Table S2: Primers for auxin responsive genes and *ACTIN2*.
