# Supplementary material for: Glutathione Enhances Auxin Sensitivity in Arabidopsis Roots
Source: Biomolecules. 2020 Nov 13;10(11):1550. doi: 10.3390/biom10111550 (PMC7697393; doi:10.3390/biom10111550)
Supplement: Supplementary file 1 [file biomolecules-10-01550-s001.zip › FigureS2.pdf]

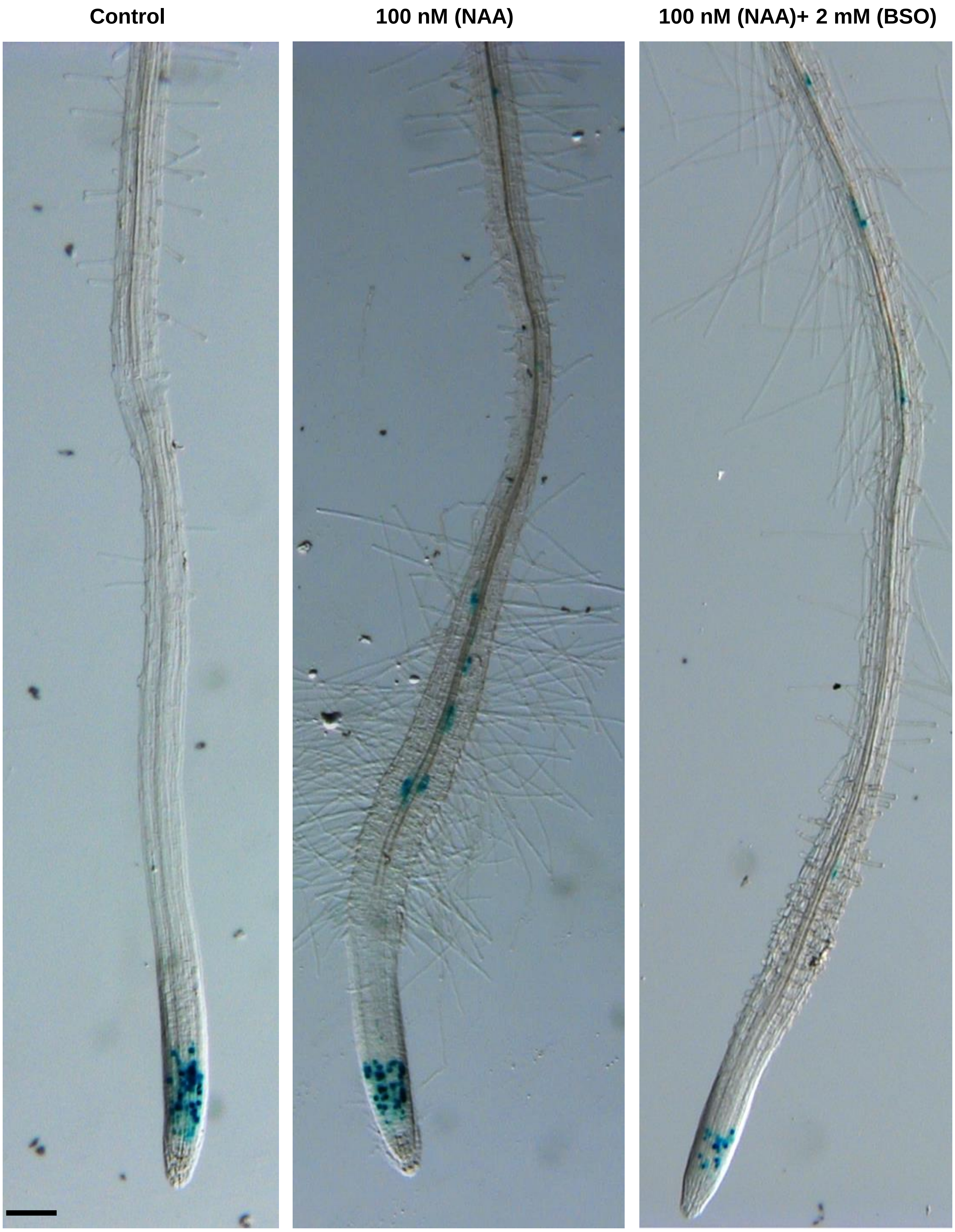


Figure S2: BSO prevents NAA-induced activation of the cell division in the distal pericycle zone. Five-day-old roots were treated with 100 nM NAA or 100 nM NAA+ 2 mM BSO for 36 hours. Scale bar is 100 µm. CyCB1::GUS was detected.
