# Supplementary material for: Glutathione Enhances Auxin Sensitivity in Arabidopsis Roots
Source: Biomolecules. 2020 Nov 13;10(11):1550. doi: 10.3390/biom10111550 (PMC7697393; doi:10.3390/biom10111550)
Supplement: Supplementary file 1 [file biomolecules-10-01550-s001.zip › Table_Suppl.docx]

**Table S1. Root length in *rml1* mutant and *rml1* mutants treated with 1 mM GSH. Three-day-old *rml1* seedlings were treated with 1 mM GSH or without (mock treated) during 3 days. Means ± SDs are presented.**

|  | **rtl1 (n=5)** | **rtl1 + 1 mM GSH (n=8)** |
| --- | --- | --- |
| **Root length (mm)** | **0.77 ± 0.13** | **4.72 ± 0.23** |

**Table S2. Primers for auxin responsive genes and actin**

|  | **Forward** | **Reverse** |
| --- | --- | --- |
| PIN1 | CTGGTCCCTCATTTCCTTCA | GCCATGAACAACCCAAGACT |
| AUX1 | TTGGGTTCGGTGGATGGGCT | AAGCGGCGACCGGGGCATGT |
| IAA2 | CGACGCTCCTGCTCTAGACT | AAAACCCCGAAGTTTCGTCT |
| IAA19 | GTGTGGCCTTGAAAGATGGT | TGAACCAGCTCCTTGCTTCT |
| ACT2 | CGCTATGTATGTCGCCA | CTTGCCCATCGGGTAA |
