# Supplementary figures and images for: Glutathione Enhances Auxin Sensitivity in Arabidopsis Roots
Source: Biomolecules. 2020 Nov 13;10(11):1550. doi: 10.3390/biom10111550 (PMC7697393; doi:10.3390/biom10111550)

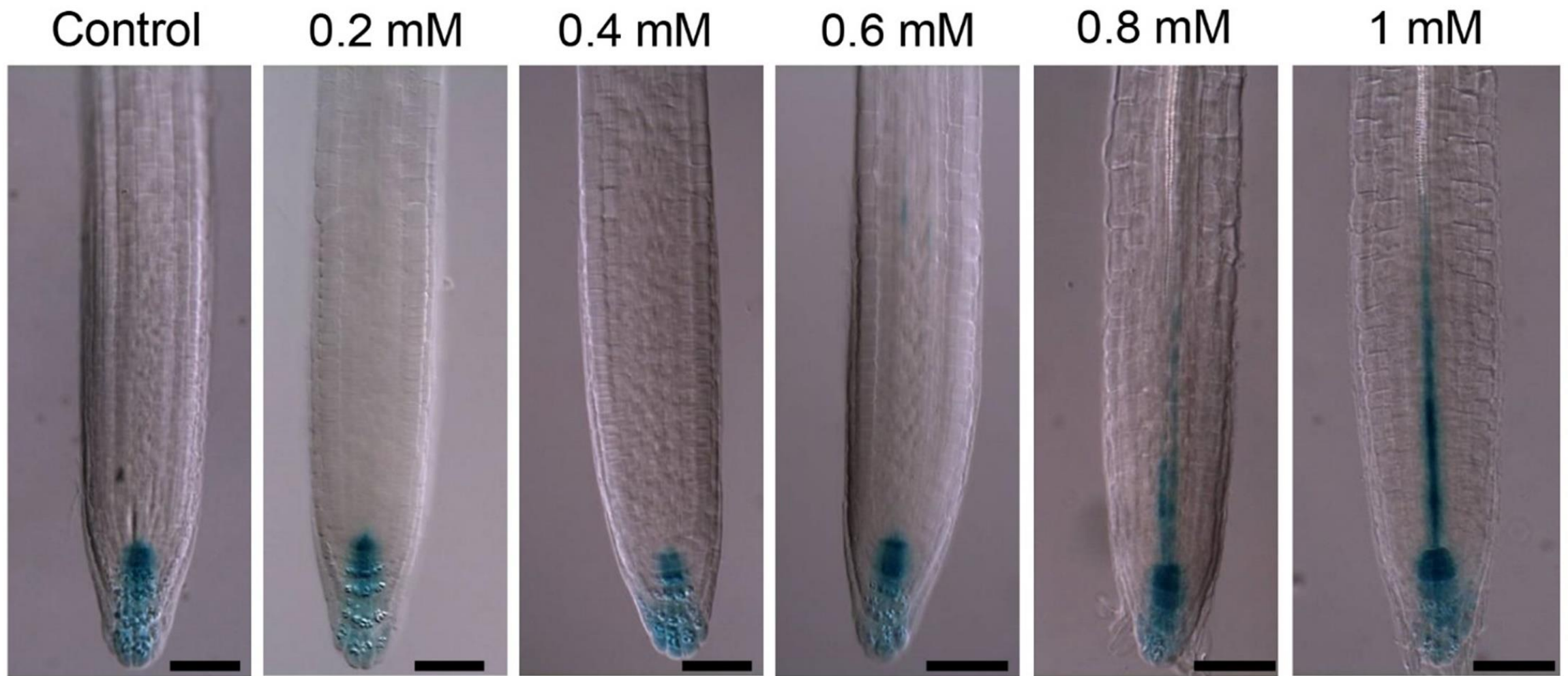

Figure S1: The effect of BSO concentrations on the DR5::GUS signal in Arabidopsis roots.

Supplement: Supplementary file 1 [file biomolecules-10-01550-s001.zip › FigureS1.pdf]
